# Studies on semantic priming effects in right hemisphere stroke: A systematic review

**DOI:** 10.1590/S1980-57642013DN70200004

**Published:** 2013

**Authors:** Juliana de Lima Müller, Jerusa Fumagalli de Salles

**Affiliations:** 1 Doctoral student at the Institute of Psychology, Federal University of Rio Grande do Sul, Porto Alegre RS, Brazil.; 2 Professor at the Institute of Psychology, Federal University of Rio Grande do Sul, Porto Alegre RS, Brazil. Coordinator of the Núcleo de Estudos em Neuropsicologia Cognitiva – Neurocog.

**Keywords:** priming, semantics, stroke, cerebrum

## Abstract

The role of the right cerebral hemisphere (RH) associated with semantic priming effects (SPEs) must be better understood, since the consequences of RH damage on SPE are not yet well established.

**OBJECTIVE:**

The aim of this article was to investigate studies analyzing SPEs in patients affected by stroke in the RH through a systematic review, verifying whether there are deficits in SPEs, and whether performance varies depending on the type of semantic processing evaluated or stimulus in the task.

**METHODS:**

A search was conducted on the LILACS, PUBMED and PSYCINFO databases.

**RESULTS:**

Out of the initial 27 studies identified, 11 remained in the review. Difficulties in SPEs were shown in five studies. Performance does not seem to vary depending on the type of processing, but on the type of stimulus used.

**CONCLUSION:**

This ability should be evaluated in individuals that have suffered a stroke in the RH in order to provide treatments that will contribute to their recovery

## Abstract (pt)

O papel do hemisfério cerebral direito (HD) associado aos efeitos de *priming* semântico (EPS) deve ser mais bem compreendido, pois as consequências de uma lesão no HD nos EPS ainda não são bem estabelecidas.

**OBJETIVO:**

Esse artigo tem como objetivo investigar estudos que analisaram EPS em pacientes acometidos por acidente vascular cerebral (AVC) no HD através de uma revisão sistemática da literatura, verificando-se se há déficits nos EPS e se o desempenho varia dependendo do tipo de processamento semântico avaliado ou dos estímulos utilizados na tarefa.

**MÉTODOS:**

Pesquisa nas bases de dados LILACS, PUBMED e PSYCINFO foram conduzidas.

**RESULTADOS:**

Vinte e sete estudos foram identificados, dos quais 11 permaneceram na revisão. Dificuldades nos EPS foram encontradas em cinco estudos. O desempenho não parece variar dependendo do tipo de processamento, mas do tipo de estímulo utilizado.

**CONCLUSÃO:**

Essa habilidade deve ser avaliada em indivíduos que sofreram um AVC no HD, fornecendo tratamentos que contribuirão a sua recuperação.

## INTRODUCTION

Priming is related to the facilitating effects of antecedent events (primes) on subsequent performance (responses to targets). This relates to perfecting the capacity of detecting or identifying words, objects or figures after a recent experience with them.^1^ The semantic priming effect (SPE), having a relation in meaning or context between the prime and the target, occurs when a word, which is preceded by another semantically related word, is processed faster and more accurately^2^ compared to control conditions. This is a cognitive phenomenon used to investigate the structure of semantic memory (general and organized knowledge of the world) and the mental representations of the word meanings and their interrelations in an implicit (indirect) manner.^3^ For a better understanding of the phenomenon, see Neely^4^ and McNamara.^5^

Semantic priming may be automatic or strategic.^5^ Automatic processes are involved when the Stimulus Onset Asynchrony (SOA) is short (less than 300 ms), when there is a low proportion of pairs of related words, and when the instructions to the participants do not mention the existence of pairs listed in the task. The spreading activation theory best explains this type of semantic priming. This theory postulates that the semantically related nodes are stored in the form of networks, and the activation of the node of the prime spreads out in the network to words that have a semantic relationship to it.^4,5^

On the other hand, controlled (strategic) processes are associated with SOAs of over 300 ms, an increased proportion of related pairs, and when the instructions include information on the existence of pairs of words listed in the experiment. The theories that explain controlled processing, in which attention processes involved, are the so-called expectancy theories.^3,4^ It is further observed that there are contradictions in relation to the value of the SOA in automatic processes: less than 300 ms,^4^ equal to or less than 200 ms^5^ and less than 150 ms.^6^

The SPE has been reported in different experimental tasks and has demonstrated aspects of the organization of the meaning of the words in semantic memory.^7^ Research evaluating the SPE in healthy participants through the divided visual field technique shows that the right cerebral hemisphere (RH) is important for maintaining the accessibility of closely and distantly related word meanings, while the left cerebral hemisphere (LH) maintains just the most closely related word meanings.^8^ Also, through studies with individuals without brain damage and using the divided visual field technique, Beeman et al. (1994)^9^ proposed the coarse coding hypothesis, which postulates that the RH undertakes a general/superficial analysis of the context while the LH selects the appropriate interpretation and executes a fine analysis. Thompkins and Lehman (1998)^10^ suggested a suppression deficit in patients with right hemisphere brain damage (RHD) in which the deviating performance with ambiguous words is related to difficulties in overcoming interpretations that are initially activated, but generally become irrelevant or incompatible with the context.

Another point to consider is that researchers indicate that the RH is involved in controlled semantic priming processes and the LH in automatic processes, which is shown in studies with the divided visual field technique.^11,12^ Thus, studies with healthy participants suggest that the right and left cerebral hemispheres contribute in different ways to lexical-semantic processing. The maintenance of broader activation and the processing of weakly related features seem to be associated with the RH, while rapid meaning access and the processing of close links is related to the LH.^3^

One way to investigate the importance of the RH to indirect lexical semantic processing is by evaluating the performance of post-stroke patients. The majority of these patients present neurological changes,^13^ with damage to various areas, including cognitive and communicative deficits.^14-16^ Neuropsychological changes after stroke depend on the regions affected, which tend to follow the distribution of the affected arteries, the extension of the injuries and the damaged hemisphere.^17^ A large proportion of the studies with neuropsychological and communication evaluation have focused on aphasic patients with lesions to the LH. The communicative and cognitive changes after damage to the LH are already well-known, such as deficits to language,^18,19^ memory^20,21^ and executive functions.^22^ The role of the RH for processing cognitive functions has been studied more recently than studies on damage to the LH.^23-26^ Better understanding of the neuropsychological deficits resulting from stroke in the RH is fundamental, enabling alternatives to preserve, improve and/or restore neuropsychological functions, contributing to a better quality of life in these patients.

It is known that damage in the RH may compromise lexical semantic processing in direct tasks, such as semantic judgment, lexical evocation, lexical access to ambiguous words (polysemic words) and upon supplying the definition of words.^26-30^ Therefore, it is important to highlight studies verifying whether indirect lexical semantic processing (semantic priming) can be preserved in patients suffering strokes in the RH.

Furthermore, important questions remain unanswered about the semantic priming paradigm. The mechanism by which the strength of association between words affects the nature of semantic priming within the RH must be better understood. Also, much behavioral evidence is in favor of the coarse coding hypothesis, but some data patterns are inconsistent with this view.^3^ Another question that warrants further investigation concerns the difficulties of RHD patients in interpreting ambiguous phrases/words and using contextual cues. Therefore, some questions associated with the role of the RH remain unanswered and are being investigated through different methodologies.

Thus, the aim of this article was to investigate studies that analyzed SPE in patients suffering a stroke in the RH through a systematic review, verifying whether there are deficits in SPEs, and whether performance varies depending on the type of semantic processing or stimulus in the task. The research questions were the following: [1] Are there deficits in SPEs in patients after stroke in the RH? [2] Does performance vary depending on the type of semantic processing (automatic versus controlled)? [3] Does performance vary depending on the type of stimulus in the task (mono or polysemic)?

## METHODS

A systematic review was carried out on PSYCINFO, PUBMED and LILACS databases up to September 2012 (all research published up to this date). The keywords used were "right hemisphere damage", "right hemisphere lesion", and "right hemisphere stroke", which were cross-referenced separately with the term "semantic priming". The same was done with the equivalent terms in Portuguese. The search criterion was the presence of the keywords in any field of research.

All studies resulting from the search were independently and systematically examined by two investigators according to exclusion/inclusion criteria. The exclusion criteria were as follows: theoretical research or reviews; studies that did not evaluate SPE; research involving samples without brain damage; studies not covering patients with stroke; research on samples without stroke in the RH. If these criteria were not clear from the title and abstract of each study, the investigators checked them in the methods section. Two papers were added from the author's personal records, due to the fact that the authors found two relevant publications that were not identified in the systematic review (a dissertation and a book chapter).

## RESULTS

None of the studies were found on LILACS database. Twenty-one studies were found on PSYCINFO, though only nine evaluated semantic priming in patients with strokes in the RH.^31-39^ The others were not included in this review for being theoretical studies,^40,41^ not covering patients with stroke,^42-45^ investigating only patients with damage to the LH,^46,47^ individuals without brain damage^48,49^ or syllabic priming.^50,51^

Out of 15 studies retrieved from the search undertaken on PUBMED, only six were related to semantic priming in patients with strokes in the RH.^32,33,35-37,39^ The six studies selected had also already been included in the search carried out on PSYCINFO. Out of the studies that did not cover semantic priming in individuals suffering from stroke, five had already been found in the previous search. The other four papers were not included in this study because they investigated only patients with damage to the LH^52^ or evaluated other types of priming, such as emotional,^53^ perceptual^54^ and abstract priming for figures.^55^

Besides the studies selected from the databases consulted, a further two papers were added from the author's personal records.^28,56^ Therefore, 11 studies were included in total. As the studies had two important categories according to the type of stimulus (polysemic or monosemic stimuli), which aimed to evaluate different issues, they were divided into these categories.

Initially, the studies using polysemic words in tasks for resolving ambiguity of words will be presented, followed by the studies making use of monosemic stimuli in their experiments. This paper shows the stimuli as they were shown in each research article.

**Studies on lexical semantic processing with right hemisphere stroke involving polysemic words.** A Canadian study undertaken by Grindrod and Baum (2003)^32^ used the semantic priming paradigm to investigate the ability in 11 aphasic, non-fluent patients with left hemisphere brain damage (LHD), 9 individuals with RHD and 20 controls with no brain damage, in the use of information from the context of sentences for resolving ambiguity of words. Three context sentences were prepared: one sentence with an unbiased context (ambiguous) (e.g. *Before giving it to her, he looked at the CARD*), a context biased by the first meaning - the most frequent one (e.g. *After writing a long message, he looked at the CARD*) and a context biased by the second meaning - the second most frequent (e.g. *Although trying not to cheat, he looked at the CARD*). Control sentences were constructed through substitution of ambiguous words at the end of the sentence by monosemic control words. The visual targets had an associative relationship with each of the meanings of the ambiguous words (e.g. *CARD - birthday* = most frequent meaning; *CARD - poker* = second most frequent meaning). These targets were paired with experimental sentences (ambiguous or control) in each of the three contexts. Each test began with the auditory presentation of a sentence. There was a subsequent inter-stimulus interval (ISI) of 0 or 750 ms, followed by the display of a visual target on a computer screen. The participants then had to make a lexical decision on the target.

The findings of this study suggested that damage to the RH causes deficits in the use of contextual information to complete the processing for resolution of ambiguities in sentences. In the lower ISI, there was no influence of the context in the groups of patients with RHD. Individuals with RHD only activated the most frequent meaning in unbiased contexts (ambiguous) and the second most frequent meaning in biased contexts. In the long interval, (ISI) patients with RHD activated the most frequent meaning in unbiased contexts and appropriately contextualized meanings in biased contexts for the second most frequent meaning. It can be concluded that damage to the RH generated difficulties in the use of local contextual information for the resolution of ambiguities in sentences.

The second study by the same authors^33^ also involved a sample of participants with RHD (n=8), LHD (n=10) and controls with no neurological impairment (n=9), where the semantic priming task was similar to that used in the previous study. However, the context provided was a four-sentence discourse instead of a single sentence. Firstly, the subjects listened to a four-sentence discourse that ended with ambiguous words (prime) and, after an ISI of 0 or 750 ms, they made a lexical decision between the first or second meaning listed (first or second most frequent meaning), presented in visual targets. Regarding the participants with RH impairment, most of them activated the second most frequent meaning when the ISI was 0 ms, and the most frequent meaning with an ISI of 750 ms (regardless of the context). However, the effects were not significant as a group. The conclusions were similar to the previous study, suggesting that RHD can cause loss in the use of context and leads to activation of the word meanings based on the frequency of the meaning of the target.

In another Canadian study, Klepousniotou and Baum (2005)^35^ investigated people with RHD (n=8), aphasic, non-fluent participants with LHD (n=9) and control participants (n=10) in the access to different meanings of three types of ambiguous words, called homonyms (cases in which a lexical item possesses two distinct and unrelated meanings), metonymies (there is a relation of connectedness between the senses of the word) and metaphors (there is a relation of analogy between the senses of the word). They used an auditory semantic priming paradigm, in which ambiguous words were incorporated into dominant (e.g. *The core of the atom is the nucleus* - metaphor) or subordinate prime sentences (e.g. *Undoubtedly, Tim is the company's nucleus* - metaphor). These sentences were followed by a short (100 ms) or long (1000 ms) ISI, and words with a related dominant meaning, related subordinate meaning, unrelated words (*electron - boss - motel* - target stimuli used for the example phrases below) or pseudowords (target stimulus). These had to be processed to carry out a lexical decision task related to the target word.

The participants with RHD showed difficulty in the use of the context mainly in the ISI of 1000 ms, while the presence of a biased context did not influence activation standards. They had SPEs with the dominant and subordinate meanings (in the homonyms and metonymies), regardless of the biased context and ISI. Patients with RHD also exhibited difficulties in the activation of subordinate meanings of metaphors (there were no SPEs in the subordinate targets in comparison with the unrelated targets), suggesting a selection problem with figurative meanings. With this study, Klepousniotou and Baum (2005)^35^ suggested that damage to the RH gives rise to deficits that suppress the alternative meanings of ambiguous words which become incompatible with the context, stating that RHD may affect the processing of polysemic words.

Another study by the same authors^36^ also investigated abilities in aphasic participants with LHD (n=10), people with RHD (n=8) and healthy controls (n=10) in the access to multiple meanings of homonyms, metonymies and metaphors. As beforehand, an auditory semantic priming paradigm and a lexical decision task were used, although in this study the words were not related to any context. Klepousniotou and Baum (2005)^36^ used homonyms, metonymies and metaphors as primes followed by three types of target words, after a short (100 ms) and long (1000 ms) ISI: [1] word related to a dominant meaning (e.g. *grass*); [2] word related to a subordinate meaning (e.g.* mile*); or [3] unrelated target words (e.g. *sin*) - possible targets of the prime "*yard*" (homonym).

Significant group effects were not found and, for both ISIs, responses to the dominant and subordinate targets were facilitated with relation to the unrelated targets in the conditions involving homonyms and metonymies. However, in the conditions in which metaphors occur, only targets related to the dominant meaning were facilitated. The researchers stated that the results obtained contradicted the suppression deficit hypothesis and the coarse semantic coding hypothesis because patients with RHD may access multiple meanings for ambiguous words and present intact processing abilities, at least at the level of single words.

The four studies described above considered patients with damage in different regions of the RH. Communicative deficits after stroke (problems with inferencing and figurative language, for example) were reported in the studies, but only because of the sample characterization. The discussion of these studies did not consider if patients with deficits in the use of contextual information or in the activation of subordinate meanings for metaphors in the processing of ambiguous words also had pragmatic difficulties evaluated through direct tasks.

Therefore, out of the four studies involving ambiguous words, the majority (three of them) showed deficits on SPEs in patients with RHD^32,33,35^ and this was seen in both automatic (short ISIs) and controlled processing (long ISIs). Damage to some areas of this hemisphere appeared to be associated with deficits in the use of contextual information in the processing of ambiguous words. Only one study showed preserved SPEs after RHD, considering automatic and controlled processing.^36^ However, the study in question did not evaluate the use of context in the lexical decision task, which is an ability that seems to be associated with the RH and could be altered after damage in this hemisphere.^3^

**Studies on lexical semantic processing with right hemisphere stroke involving monosemic pairs of stimuli.** The study conducted in the United States by Tompkins et al. (2008)^39^ investigated whether deficits in the processing of secondary and/or distantly related meanings, as typically observed in the study of homonyms in patients with RHD, extended to peripheral semantic properties weakly related to monosemic nouns. A total of 28 adults with unilateral RHD resulting from a stroke episode and 38 adults without brain damage participated in the study. The participants heard spoken sentences that ended with a monosemic noun (e.g. *He has an apple*), which was the prime. Each sentence was followed by a target (spoken word), using two ISIs (175ms and 1000 ms). The targets were composed of three types of real words: semantic properties of the nouns in the sentences that were: [a] compatible (related-compatible; e.g. *crunchy*); or [b] incompatible (related-incompatible; e.g. *rotten*) with the dominant mental image of the noun; and [c] unrelated words (e.g. *mermaid*). A lexical decision task was used to verify the initial activation and maintenance of the activation for these semantic properties of weak relations. The results for accuracy indicated SPE in both types of peripheral properties (related-compatible and related-incompatible) in the group with RHD with an ISI of 175ms. This group did not show a priming effect at the ISI of 1000 ms, which may be associated with a rapid drop/maintenance of the activation of distantly related properties.

On the other hand, the Canadian study by Gagnon et al. (1994)^28^ considered moderately and weakly associated words (distantly related) and reported different results from the previous research. These authors conducted an experiment with patients with RHD (n=10) and healthy controls (n=10) through a lexical decision task. The primes were formed by words or a series of four "x" stimuli (neutral conditions) and the targets by words or pseudowords, both presented visually. SOAs of 300 ms and 1000 ms were used. The researchers showed that patients with RHD can have preserved SPE in automatic (short SOA) and controlled processing (long SOA).

Müller (2012),^56^ in Brazil, also evaluated SPEs through a visual lexical decision task but with strongly related words and 500 ms SOA. The sample was composed of patients with RHD (n=11) and healthy controls (n=11). In the experiment, the stimuli were formed by prime-target pairs of words semantically related (e.g. *noite-dia*), semantically unrelated (e.g. *sol-luva*) or pairs with a pseudoword target (e.g. *sangue-rídia*). A group study and a case series investigation were performed. The group comparison results showed that the group with RHD presented SPEs. However, the case series study with the clinical sample found heterogeneity in the performance of the patients on the semantic priming task, since a part of the sample showed preserved SPE (72%) and the remainder, (28%) impaired SPE.

The North American study by Henik et al. (1993)^34^ investigated SPE in patients with lesions in the right (n=9) or left (n=19) brain hemispheres only, anterior or posterior, and a control group (n=12). In the semantic priming experiment, pairs of related prime-target words (e.g. *DOCTOR-NURSE*), unrelated prime-target words (e.g. *BREAD-NURSE*) and pairs with a pseudoword target (e.g. *DOCTOR-SURNE*) were used and presented visually. The sort of relationship between the pairs of related prime-target was not specified. Individuals carried out a lexical decision task. SOAs of 250 ms and 1850 ms were manipulated. Patients with RHD had SPE preserved in both SOAs.

In another North American research conducted by Shah and Baum (2006)^38^ prosodic processing and semantic priming were evaluated concomitantly. The investigation was undertaken to examine the ability of individuals with damage to the LH (n=10), RH (n=9) and controls without brain damage (n=14) to perceive lexical stress cues and to map them in lexical semantic representations. The study evaluated sensitivity to the manipulation of the lexical tone in a lexical decision task that required processing of prosodic information and activation of the meaning of the word. The authors verified whether the patients were capable of using lexical tone cues to activate lexical semantic representations. Primes with highlighted correct and incorrect stressed syllables were paired with related target words (e.g. correct-*CANcer-Disease*; incorrect -*feMALE-Woman*), unrelated words (e.g. correct- *PAINter-Basis*; incorrect-*CAffeine-Hotel*) and with pseudowords (e.g. correct*beLOW-Nefius*; incorrect- *flyING-Zarfer*) to explore the implicit processing of lexical prosody. The stimuli were presented audibly with an ISI of 250 ms. According to the results, the increased sensitivity of the variation of the stress standards of the primes was demonstrated by the group with RHD (compared to the other groups). It is suggested that individuals with lesions to the RH maintain sensitivity to lexical prosody in the English language, as they presented intact prosodic processing in indirect tasks at the lexical level.

Hemispatial neglect has also been considered in priming studies with stimuli involving words that have only one meaning. In an Italian study, Làdavas et al. (1993)^37^ investigated associative semantic priming in a 63-year-old, right-handed patient with RHD and left-hand side visual neglect. The study investigated whether the information presented to the neglected visual field could be processed at the lexical-semantic level. Out of the experiments conducted, only one referred to semantic priming. In this experiment, there were pairs of related words (e.g. *canne-gatto*), unrelated pairs (e.g. *vestito-gatto*), and pairs formed by a word and a pseudo-word. The related prime-target words were of the same semantic category or the prime was a word commonly described by the target. The stimuli were presented in visual modality. A square appeared in the center of the computer screen. After an ISI of 100 ms, 300 ms or 500 ms, a prime word would appear on the left hand side of the computer screen for 200 ms (centered 5.5 degrees to the right of the square). Again, using the same ISIs, a target (word or pseudoword) was presented on the right hand side of the square and the participant made a lexical decision on the target.^37^ The patient showed SPE in the neglected space, i.e. the response to a word in the right visual field was quicker when the word was preceded by a brief presentation of a semantically associated word in the neglected field.

In a North American study, D'Esposito et al. (1993)^31^ also investigated the processing of information presented to the neglected hemisphere, evaluating 16 patients with visual neglect following stroke in the RH. These authors used a lexical decision task to evaluate SPEs in which primes that were related and unrelated to the target appeared in the left or right visual field. There was the display of a sequence of the letter "x" in the opposite field and the SOA was 600 ms. This was followed by the visual presentation of the target in the center of the screen. Two versions of the experiment were run, differing only in the nature of the prime. In one of the versions, figures of objects were used, while in the other, the name of the objects was the prime. The result indicated that the patients showed preserved SPEs.

The studies involving monosemic words also considered patients with damage in different regions of the RH. Only one study failed to specify the region of the RH stroke.^28^ Communicative deficits after stroke providing better sample characterization were reported in only some studies.^28,38,56^ It is important to emphasize that Shah and Baum (2006)^38^ did not evaluate whether their sample had difficulties in direct tasks involving prosodic processing.

Four studies performed evaluation of access to semantic knowledge using direct as well as indirecttasks. _28,^31,37,56^_ Gagnon et al. (1994)^28^ showed that patients with RHD may present preserved SPE in automatic and controlled processing, yet deficits in semantic judgment task. Müller (2012)^56^ also evaluated SPEs and lexical-semantic processing with direct tasks (semantic judgment and verbal fluency tasks), finding four types of performance: performance preserved on SPE and direct tasks; performance impaired on SPE and direct tasks; performance impaired on SPE only; and performance impaired on direct tasks only.

D'Esposito et al. (1993)^31^ used a semantic priming task and a direct task (a delayed forced-choice discrimination task). Patients showed preservation of SPE, but difficulty in direct tasks. Làdavas et al. (1993)^37^ evaluated SPE and lexical semantic processing through direct tasks (reading, semantic judgment, lexical decision and detection of signals) and also found that the patient had preserved SPE, but difficulty in direct tasks.

Thus, out of the seven studies involving monosemic words, only two showed impaired SPEs in individuals with RHD. One of these studies observed deficits considering an ISI of 1000 ms,^39^ while the other indicated impaired SPEs in 28% of the sample considering an SOA of 500 ms.^56^ Both studies seem to evaluate controlled processing. The remaining studies showed preserved SPEs, considering automatic and/or controlled processing.

As for the type of stimulus (mono or polysemic), it could be seen that out of the four studies involving polysemic stimulus, three showed deficits on SPEs in patients with RHD,^32,33,35^ regardless of the type of processing evaluated. However, out of seven studies involving monosemic words, only two showed impaired SPEs in patients with RHD,^39,56^ and the deficit was associated only with controlled processing.

## DISCUSSION

From the analysis of all of the studies covered in this literature review, we can highlight several points. Regarding the first research question, it was possible to verify that difficulties associated with SPEs were shown in five out of eleven studies.^32,33,35,39,56^

The results profile was heterogeneous. In general, performance does not seem to vary consistently depending on the type of processing (whether automatic or controlled, second research question), since out of the studies involving ambiguous words, three showed deficits on SPEs in patients with RHD,^32,33,35^ which was seen in both automatic (short ISIs) and controlled processing (long ISIs). Out of the studies involving monosemic words, only two showed impaired SPEs in individuals with RHD,^56,39^ evaluating controlled processing. However, four other studies showed preserved SPEs^28,31,34,37^ also evaluating controlled processing, indicating that the difficulty may not be associated with type of processing.

On the other hand, the performance of individuals with RHD seems to vary depending on type of stimulus (third research question). Impaired SPEs were shown in only two^56,39^ out of seven studies with tasks involving monosemic words, while this kind of performance was observed in the majority of the (three out of four)^32,33,35^ studies with polysemic words.

In the studies with monosemic words, most authors observed that lexical semantic processing was preserved in patients with RH stroke,^28,31,34,37^ when the SOA or ISI was short (≤300 ms) or long (≥ 500 ms). However, Tompkins et al. (2008),^39^ in a group study, and Müller (2012),^56^ through a case series investigation, did not find SPEs at least in part of the samples.

The study of Tompkins et al. (2008)^39^ did not show SPEs in patients with stroke in the RH with an ISI of 1000 ms. However, these same effects were not observed in controls with this ISI. This result suggests that the performance of these patients may not be the result of a deficit in lexical semantic processing and should be further investigated. Müller (2012)^56^ also used monosemic words with a SOA of 500 ms. It was suggested in the study that patients with RHD may present difficulties associated with access to strongly related words. Frishkoff (2007),^57^ in a study with healthy participants, indicated that SPEs in the RH are associated with strongly related word pairs. Thus, these results showed that the RH may play an important role in the processing of strongly semantic relationships between words. An important issue of note is that deficits on SPEs in research with monosemic words may have been masked by group studies, as was shown by Müller (2012).^57^

In research with polysemic words, most studies indicated that SPEs were impaired in individuals with RHD,^32,33,35^ considering both short (≤100 ms) and long ISI (≥ 750 ms). These difficulties were associated with the following: use of contextual information when processing ambiguous words;^32,33,35^ activation of subordinate meanings for metaphors, with the suppression of alternative meanings for ambiguous words.^35^

Evidence has shown that the RH's contribution increases as use of complex, natural language increases.^58^ Activation in this hemisphere is associated with the search of contextual relevance of linguistic stimuli,^59^ which could be related to the difficulty in the use of contextual information when processing ambiguous words indicated by some studies.^32,33,35^ It seems that the role of this hemisphere is more evident when the experiment addresses aspects of natural language, as suggested by Kahlaoui et al. (2008).^3^

Tompkins, Baumgaertner, Lehman, and Fassbinder (2000)^60^ indicated the ability of the RH to make effective use of contextual cues to suppress inappropriate meanings. Therefore, the deficit in activation of subordinate meanings for metaphors emphasized by Klepousniotou and Baum (2005)^35^ may be associated with this fact. Furthermore, Chiarello and Richards (1992)^8^ suggested that the RH is important for maintaining the accessibility of both distantly and closely related word meanings. Beeman and Chiarello (1998)^61^ confirmed this idea and pointed out the ability of this hemisphere to sustain more remote and distant semantic associates. Beeman et al. (1994)^9^ also proposed the coarse coding hypothesis and according to this, the RH is responsible for activating several meanings and many features of the word, as features that are distantly related to the input word.

Through this literature review, it was possible to verify that the studies encountered in the search, besides being small in number, used very different stimuli, such as ambiguous words, monosemic words, sentences or words, figures, prosodic stimuli, etc. The form of stimuli presentation also differed between studies (visual, auditory or both auditory and visual). Four studies used auditory tasks,^35,36,38,39^ while five used visual tasks.^28,31,34,37,56^ Two other studies presented the prime in an auditory manner and the target visually.^32,33^

Other factors could also be interfering with the diversity of findings, for example, the experimental task, the stimulus selection criteria, the sample selection criteria, the sample heterogeneity, the small clinical groups and the SOA or ISI used in the semantic priming experiment (controlled or automatic semantic processes). It is also essential to emphasize that not showing the SOA of a study can be problematic for identifying the processes involved (automatic or controlled).

In conclusion, the present systematic review suggests that RHD may give rise to difficulties in SPEs associated with the following abilities: use of contextual information when processing ambiguous words;^32,33,35^ activation of subordinate meanings for metaphors, with the suppression of alternative meanings for ambiguous words;^35^ access to semantic properties distantly related to their corresponding lexical items;^39^ access to strongly related words.^56^ These abilities should be evaluated in individuals that have suffered a stroke in the RH. Neuropsychological rehabilitation should be considered in patients with deficits in lexical semantic processing,^62^ contributing to their recovery and quality of life.

Another issue to consider is the observation that, in some of the studies, abilities associated to semantic processing were preserved in the patients post-stroke. Right hemisphere damage was associated to preservation in access to multiple meanings for ambiguous words when there was no use of context.^36^ Also, the majority of the studies with monosemic words showed preserved SPE.^28,31,34,37^ However, it is possible that deficits on SPEs in research with monosemic words have been masked by group studies. Therefore, the development of case series investigations is important to further understanding of SPEs in post-stroke RH. It is also important to evaluate lexical-semantic processing after RHD using direct and indirect tasks, verifying the existence of possible associations and dissociations.

Future studies should consider similar methodologies to the existing ones, allowing more accurate comparisons among results and providing deeper understanding of the RH in lexical semantic processing. SPEs after LHD should also be investigated through literature reviews, as some authors have been considering.^63^
